# Drug Sensitivity Testing in Cytoreductive Surgery and Intraperitoneal Chemotherapy of Pseudomyxoma Peritonei

**DOI:** 10.1245/s10434-015-4675-0

**Published:** 2015-07-21

**Authors:** Kathrine Bjersand, Haile Mahteme, Inger Sundström Poromaa, Håkan Andréasson, Wilhelm Graf, Rolf Larsson, Peter Nygren

**Affiliations:** Department of Women’s and Children’s Health, Uppsala University, Uppsala, Sweden; Department of Surgical Sciences, Uppsala University, Uppsala, Sweden; Department of Medical Sciences, Uppsala University, Uppsala, Sweden; Department of Immunology, Genetics and Pathology, Uppsala University, Uppsala, Sweden

## Abstract

**Background:**

Cytoreductive surgery (CRS) and intraperitoneal chemotherapy (IPC) is an established therapy for pseudomyxoma peritonei (PMP). However, the role of IPC is unclear. By ex vivo assessment of PMP tumor cell sensitivity to cytotoxic drugs, we investigated the basis for IPC drug selection and the role of IPC in the management of PMP.

**Methods:**

Tumor cells were prepared by collagenase digestion of tumor tissue from 133 PMP patients planned for CRS and IPC. Tumor cell sensitivity to oxaliplatin, 5FU, mitomycin C, doxorubicin, irinotecan, and cisplatin was assessed in a 72-h cell-viability assay. Drug sensitivity was correlated to progression-free survival (PFS) and overall survival (OS).

**Results:**

Samples from 92 patients were analyzed successfully. Drug sensitivity varied considerably between samples. Peritoneal mucinous carcinomatosis (PMCA), compared with PMCA intermediate or disseminated peritoneal adenomucinosis, was slightly more resistant to platinum and 5FU and tumor cells from patients previously treated with chemotherapy were generally less sensitive than those from untreated patients. Multivariate analysis showed patient performance status and completeness of CRS to be prognostic for OS. Among patients with complete CRS (*n* = 61), PFS tended to be associated with sensitivity to mitomycin C and cisplatin (*p* ≈ 0.06). At the highest drug concentration tested, the hazard ratio for disease relapse increased stepwise with drug resistance for all drugs.

**Conclusions:**

Ex vivo assessment of drug sensitivity in PMP provides prognostic information. The results suggest a role for IPC as therapeutic adjunct to CRS and for individualization of IPC by pretreatment assessment of drug sensitivity.

**Electronic supplementary material:**

The online version of this article (doi:10.1245/s10434-015-4675-0) contains supplementary material, which is available to authorized users.

Pseudomyxoma peritonei (PMP) is a rare tumor disease characterized by disseminated mucus and mucinous tumor tissue implants on the peritoneal surfaces, now considered to originate from the appendix.^1^ By use of cytoreductive surgery (CRS) combined with intraperitoneal chemotherapy (IPC), as introduced by Sugarbaker, with the IPC now mostly being hyperthermic (HIPEC), the prognosis has improved.^2^ Thus, experienced centres report a 5-year overall survival (OS) in the range of 70–95 % compared with 30–40 % often reported for the strategy of debulking surgery.^3^–^5^ The main factors associated with favorable prognosis are complete CRS, low tumor load, and low histological grade.^5^

Systemic chemotherapy alone as treatment of PMP has not been extensively investigated but seems poorly active in this disease.^6^ However, the role of the chemotherapy part of the CRS and IPC treatment package in PMP is unclear. Thus, the effect of CRS with or without IPC has not been directly compared and long-term survival has been reported with CRS alone.^5^,^7^,^8^

In contrast, IPC provides clinical benefit as adjunct to CRS in peritoneal carcinomatosis from gastric and ovarian cancer.^8^,^9^ Furthermore, IPC in PMP differs between treatment centres in terms of drug selection, dosing, and timing of the IPC.^8^,^10^ Thus, IPC as part of treatment of PMP is in need of further investigation to define its role and provide a basis for how to optimize this resource demanding treatment step.

We used a short-term ex vivo assay to evaluate the tumor cell sensitivity to cytotoxic drugs in samples from PMP patients undergoing CRS and IPC. The goals were to provide information on the pattern of drug activity in PMP and to correlate the ex vivo drug sensitivity pattern to the clinical outcome.

## Methods

### Patients and Tumor Samples

A total of 133 patients scheduled for CRS and HIPEC for PMP at the Department of Surgery, Uppsala University Hospital between May 2006 and December 2011, and from which a tumor sample for ex vivo assessment of drug activity was obtained, formed the basis for the study. Tumor sampling was performed intraoperatively prior to HIPEC, which consisted of 30–35 mg/m^2^ of mitomycin C, 100 mg/m^2^ of cisplatin combined with 15 mg/m^2^ of doxorubicin *or* 360 mg/m^2^ of both irinotecan and oxaliplatin.^4^ Tumor sampling and data collection was based on patient informed consent and approved by the Regional Ethical Review Board in Uppsala (Dnr 2007/237). None of the patients had adjuvant systemic chemotherapy following CRS and HIPEC.

Tumor histopathology was classified as disseminated peritoneal adenomucinosis (DPAM), peritoneal mucinous carcinomatosis (PMCA), or PMCA with intermediate features.^11^ Tumor load was assessed as the Peritoneal Cancer Index (PCI) at time of surgery.^12^ Residual disease after a maximal surgical effort was quantified according to the completeness of cytoreduction score (CC). CC scores 0 (no macroscopic tumor left) and 1 (residual tumor <0.25 cm) were considered as complete cytoreduction.^13^

### Ex Vivo Assessment of Drug Sensitivity

The tumor specimen was kept in buffer at 6 °C until preparation. Tumor cells were prepared by collagenase digestion as described.^14^ The cells obtained were mostly single cells or small cell clusters with ≥90 % viability and with <30 % contaminating nonmalignant cells, as judged by morphological examinations of May-Grünwald-Giemsa-stained cytocentrifugate preparations.

The drugs used for HIPEC (see above) were tested ex vivo. In addition, 5FU, an established drug in gastrointestinal cancer treatment, was included. All drugs were from commercially available clinical preparations. The drugs were tested at three tenfold dilutions from the maximal concentration (μM) of 100 for cisplatin, 100 for oxaliplatin, 10 for doxorubicin, 1000 for 5FU, 100 for mitomycin C, and 1000 for irinotecan. The drug concentrations used ex vivo are chosen empirically to produce concentration—response curves allowing for extraction of 50 % inhibitory concentrations (IC_50_), i.e., the drug concentration producing a cell survival of 50 % compared with an unexposed control. The maximal concentrations used ex vivo are close to *C*_max_ achievable during IPC for most drugs.^15^ 384-well microplates (Nunc) were prepared with 5-μl drug solution at 10× the final drug concentration using the pipetting robot BioMek 2000 (Beckman Coulter). The plates were then stored at −70 °C until further use.

The semiautomated fluorometric microculture cytotoxicity assay (FMCA) was used to assess drug sensitivity.^16^ Briefly, tumor cells from patient samples (5000 cells/well in 45 μl culture medium RPMI 1640 (supplemented with 10 % foetal calf serum, glutamine and antibiotics) were seeded in the drug-prepared 384-well plates using the pipetting robot Precision 2000 (Bio-Tek Instruments Inc., Winooski, VT). Three columns without drugs served as controls and one column with medium only served as blank.

The culture plates were incubated at 37 °C in humidified atmosphere containing 95 % air and 5 % CO_2_. After 72 h incubation, the culture medium was washed away and 50 μl/well of a physiological buffer containing 10 μg/ml of the vital dye fluorescein diacetate (FDA) were added to control, experimental, and blank wells. After incubation for 30–45 min at 37 °C, the fluorescence from each well was read in a FluoroScan 2 (Labsystems OY, Helsinki, Finland).

Quality criteria for a successful assay were: ≥70 % tumor cells in the cell preparation before incubation and/or on the assay day, a fluorescence signal in control cultures of ≥5 *x* mean blank values, and a coefficient of variation of cell survival in control cultures of ≤30 %. The results obtained by the viability indicator FDA are calculated as survival index (SI), defined as the fluorescence of the test expressed as a percentage of control cultures, with blank values subtracted.

### Patient Data and Follow-Up

Clinical data relevant for the study were retrieved from the patient files. Patients with complete cytoreduction were followed for progression-free survival (PFS) by assessment of serum tumor markers (CEA, CA19-9, CA 125, and CA 72.3) every 3 months and with CT scan of abdomen and thorax every 6 months for 3 years and then every 12 months, for another 2 years. An increase in a tumor marker ≥25 % triggered a CT scan for verification of new lesions consistent with PMP relapse. Overall survival (OS) was assessed from registry data up to February 2014. Data on treatment following relapse was incomplete and indicated individualized approaches used. This is expected to affect the OS observed probably making this endpoint poorly associated to the IPC (see “Results” section).

### Data Evaluation and Statistics

IC_50_ was calculated using non-linear regression to a standard sigmoidal dose–response model in GraphPad Prism version 5 for Mac (GraphPad Software, San Diego, CA). Alternatively, sample sensitivity was scored according to the SI at the highest cytotoxic drug concentration used ex vivo. In this case, low drug resistance (LDR) was defined as a SI below the median, intermediate drug resistance (IDR) as a SI between the median and median plus two standard deviations (SDs), and extreme drug resistance (EDR) as a SI above median plus two SDs based on all samples investigated ex vivo.^16^,^17^

Statistical inferences between several means were performed by one-way ANOVA with Tukey HSD post-hoc tests. The prognostic importance of clinicopathological variables and ex vivo drug sensitivity for OS and PFS was assessed in a Cox regression model. In the model on OS only univariate results with *p* < 0.2 were included in the final multivariable analysis. Analyses on PFS were adjusted for WHO performance status, histopathological subtype, and tumor load. The level of significance for all statistical tests was set to *p* < 0.05. Data are presented as mean ± SD unless otherwise stated.

## Results

A successful ex vivo assay fulfilling the quality criteria was obtained from 92 tumor samples (69 %) and data from these patients were included for analysis in the study. Mucin-rich tumor samples, often of the DPAM subtype, dominated among samples not possible to run in the assay due to difficulties to recover a sufficient number of epithelial cells when a lot of mucin was present during cell preparation. The majority of patients had a histopathology of DPAM (*n* = 57), whereas 24 had PMCA and 11 patients had a PMCA intermediate histology (Table 1). A majority of patients, 64 %, had previously been treated systemically and/or locally with chemotherapy for PMP. In 61 patients (66 %) CC 0–1, i.e., complete cytoreduction was achieved at CRS. Eighty patients, including 59 of the 61 patients with complete cytoreduction, received HIPEC, most commonly single drug mitomycin C (*n* = 56), combinations of cisplatin and doxorubicin (*n* = 17), or irinotecan and oxaliplatin (*n* = 7).

**Table 1:** Clinical characteristics of the pseudomyxoma peritonei samples successfully analyzed ex vivo (*n* = 92)

Age, year, mean (range)	56 (24–78)
BMI, kg/m^2^, mean (range)	25 (19–38)
Male/female	47/45
Histopathology	
DPAM	57 (62 %)
PMCA intermediate	11 (12 %)
PMCA	24 (26 %)
Prior chemotherapy	
No	59 (64 %)
Yes	33 (36 %)
PCI score^a^	
1–10	9 (10 %)
11–20	13 (14 %)
21–39	69 (76 %)
WHO performance status	
0	79 (86 %)
1–2	13 (14 %)
Complete cytoreductive surgery^b^	61 (66 %)
Hyperthermic intraperitoneal chemotherapy	80 (87 %)

*DPAM* disseminated peritoneal adenomucinosis, *PMCA* peritoneal mucinous carcinomatosis, *PCI* peritoneal carcinoma index, *WHO* World Health Organization

^a^Information on PCI score unavailable in one patient

^b^CC score 0–1

Drug sensitivity varied considerably between patient samples as indicated by the high SDs observed for the IC_50_ values for all drugs (Table 2). Samples obtained from patients previously exposed to cytotoxic drugs were statistically significantly more resistant to all drugs tested except irinotecan. There were statistically significant differences in drug sensitivity to oxaliplatin, 5-FU, and cisplatin between the histopathological subtypes of PMP (Table 2); PMCA showed higher IC_50_ values for oxaliplatin, 5-FU, and cisplatin compared with samples of hybrid histology. Similarly, PMCA samples had higher IC_50_ for oxaliplatin than DPAM. There were no statistically significant differences in drug sensitivity between samples divided into low and high grade according to Bradley (Supplementary Table 1).^18^

**Table 2:** IC_50_ values (μM, mean ± standard deviation) for the indicated drugs in the pseudomyxoma peritonei samples (*n* = 92; IC_50_ values available in 88–92 cases depending on cytotoxic drug) according to previous chemotherapy and histopathological subtype

	Previous chemotherapy		Histopathological subtype			All PMP
	Yes *n* = 59	No *n* = 33	DPAM *n* = 57	PMCA intermediate *n* = 11	PMCA *n* = 24	*n* = 92
Oxaliplatin	47.2 ± 36.1	25.9 ± 29.4^a^	30.9 ± 32.0	16.3 ± 12.0	47.9 ± 38.8^b,c^	33.6 ± 33.5
5FU	708 ± 354	517 ± 431^a^	591 ± 436	327 ± 256	692 ± 376^c^	586 ± 414
Mitomycin C	35.0 ± 75.6	12.1 ± 16.8^a^	22.4 ± 59.6	11.4 ± 19.6	20.0 ± 20.5	20.4 ± 48.3
Doxorubicin	3.3 ± 5.6	1.5 ± 3.0^a^	2.6 ± 4.9	1.1 ± 2.0	1.5 ± 2.7	2.1 ± 4.2
Irinotecan	410 ± 755	223 ± 311	347 ± 631	1126 ± 78	244 ± 290	291 ± 522
Cisplatin	41.0 ± 35.8	25.6 ± 33.4^a^	29.8 ± 31.8	13.9 ± 11.7	42.4 ± 45.1^c^	31.1 ± 34.9

^a^Statistically significant difference from patients who received preoperative cytotoxic drug treatment, *p* < 0.05 by Mann–Whitney *U* test

^b^Statistically significant difference from DPAM, *p* < 0.05, Kruskal–Wallis followed by Mann–Whitney *U* test

^c^Statistically significant difference from PMCA intermediate, *p* < 0.05–0.01, Kruskal–Wallis followed by Mann–Whitney *U* test

Analysis of OS according to clinical variables, histopathology, and drug sensitivity were performed by uni- and multivariable Cox regression for all patients with successful ex vivo assay (*n* = 92; Supplementary Table 2). Drug sensitivity was not statistically significantly associated with OS (univariate hazard ratio range, 0.59–1.20), whereas, as expected, impaired WHO performance status and completeness of cytoreduction (no vs. yes) were associated with shorter OS (hazard ratio, 7.93 and 11.73, respectively; *p* < 0.001).

Because of the strong prognostic value of complete cytoreductive surgery, subsequent analyses on prognostic impact of ex vivo drug sensitivity were performed in patients with complete cytoreduction (*n* = 61) with PFS as the clinical endpoint. Because only five patients in this group died during follow-up, analysis on OS was not considered. Following adjustment for performance status, PCI score, and histopathologic subtype, a strong trend towards longer PFS was observed for individuals with tumors sensitive to mitomycin C and cisplatin (*p* = 0.063 and 0.062, respectively; Table 3).

**Table 3:** Univariate and multivariate Cox regression model for progression-free survival according to dichotomized drug sensitivity values (below vs. above the median IC_50_) and clinicopathological variables in pseudomyxoma peritonei patients with complete cytoreductive surgery (*n* = 61)

	Univariate hazard ratio	*p*	Multivariate hazard ratio^a^	*p*
Oxaliplatin	1.33	0.6	0.96	1.0
5-FU	1.24	0.7	0.98	1.0
Mitomycin C	0.66	0.4	0.36	0.063
Doxorubicin	1.49	0.5	1.21	0.8
Irinotecan	0.95	1.0	0.72	0.6
Cisplatin	0.54	0.3	0.36	0.062

^a^Adjusted for histopathological subtype, PCI score, and WHO performance status

**Fig. 1:**
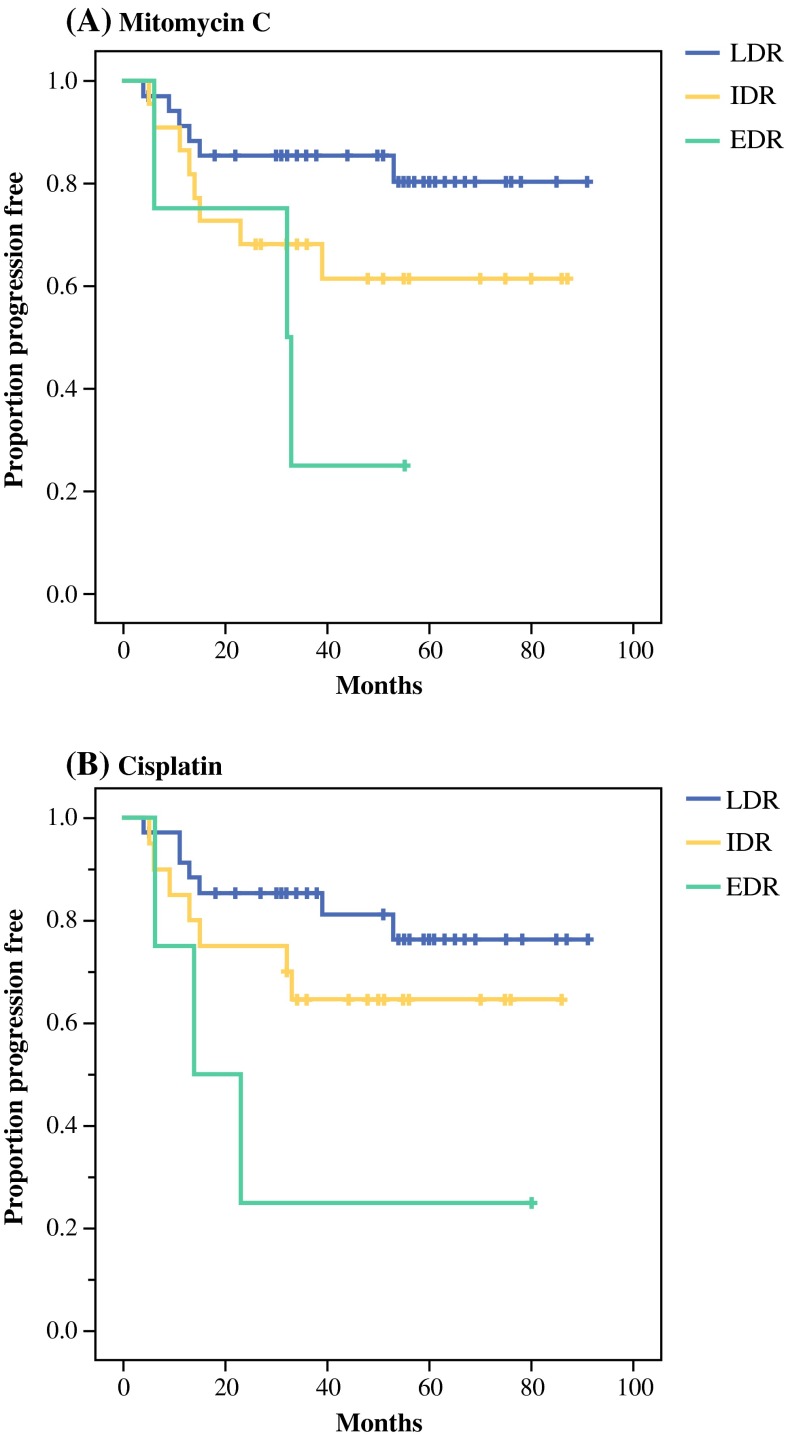
Progression-free survival in patients with complete cytoreductive surgery according to ex vivo sensitivity to mitomycin C and cisplatin categorized into low drug resistance (LDR), intermediate drug resistance (IDR), and extreme drug resistance (EDR) at the highest drug concentration tested ex vivo. Adjusted for patient performance status, histopathological subtype, and PCI score in a Cox regression model. For details on number of patients and statistical significance, see Table 4

Because very high concentrations of cytotoxic drugs are obtained locally when subjects are treated with IPC, additional analyses on drug sensitivity in relation to PFS were conducted based on the drug activity, categorized as LDR, IDR, and EDR, at the highest drug concentration used ex vivo.^15^ Following adjustment for patient performance status, histopathological subtype, and PCI score, the general pattern observed was that of a stepwise increase in risk for disease progression when going from LDR to IDR and EDR ex vivo sensitivity scores (Table 4). This was statistically significant for cisplatin and 5FU and marginally so for mitomycin C. The stepwise decrease in PFS when comparing LDR to IDR and EDR for mitomycin C and cisplatin is illustrated in Fig. 1.

**Table 4:** Univariate and multivariable Cox regression model for progression-free survival according to drug sensitivity at the highest cytotoxic drug concentration used ex vivo in pseudomyxoma patients with complete cytoreductive surgery (*n* = 61)

	*n*	Univariate hazard ratio	*p*	Multivariate hazard ratio^a^	*p*
Mitomycin C					
LDR	35	1		1	
IDR	22	2.32	0.2	3.38	0.05
EDR	4	5.19	0.05	6.00	0.05
Cisplatin					
LDR	35	1		1	
IDR	20	1.86	0.3	3.00	0.064
EDR	4	5.16	0.05	14.35	0.001
Irinotecan					
LDR	30	1		1	
IDR	26	1.38	0.6	1.53	0.5
EDR	5	1.93	0.5	1.68	0.6
5FU					
LDR	30	1		1	
IDR	26	0.52	0.3	0.55	0.4
EDR	4	3.83	0.05	4.91	0.05
Oxaliplatin					
LDR	33	1		1	
IDR	24	0.68	0.7	2.26	0.2
EDR	3	1.28	0.9	3.52	0.3
Doxorubicin					
LDR	32	1		1	
IDR	20	1.05	1.0	1.03	1.0
EDR	7	1.8	0.4	1.76	0.5

^a^Adjusted for histopathological subtype, PCI score, and WHO performance status

## Discussion

This study investigated the poorly defined role of IPC in PMP by ex vivo assessment of tumor cell sensitivity in an assay shown to reflect clinically relevant drug sensitivity in diagnostic groups as well as in individual patients.^14^,^17^,^19^,^20^ The number of patients included was quite large for this uncommon tumor type and OS was strongly associated with completeness of the CRS and patient performance status, as expected, indicating that our material and the findings are representative for PMP.^4^,^5^

The fact that the extent of CRS was strongly associated with OS, whereas ex vivo drug sensitivity was not, points to the importance of qualified surgery to achieve long-term survival in PMP. However, ex vivo drug sensitivity provided prognostic information for PFS and points to a possible impact of IPC on PFS when added to CRS. These observations are largely in line with previous findings pointing to a benefit from HIPEC for PFS but not OS as well as the possibility to achieve long-term OS with surgery alone.^4^,^5^,^21^–^23^ Still, a prolongation of PFS from IPC is considered clinically relevant, because it is reasonably associated with less disease-related symptoms and, thus, improved quality of life.

The observations indicating that IPC has limited effect and require good CRS make sense from a tumor biology point of view, because the penetrance of cytotoxic drugs into tumor tissue is very limited. Thus, no substantial effect of IPC on macroscopic tumor lesions is to be expected.^8^ Still, some effect from IPC in the presence of remaining macroscopic disease following CRS cannot be excluded. In our series, patients without complete CRS who had HIPEC (*n* = 21) had a OS of 63 versus 7 months for those who had not (*n* = 10), a difference that was statistically significant also after adjustment for performance status, histopathology, and PCI (*p* = 0.007; not shown). However, patient selection based on other prognostic factors reasonably explains most of this difference.

Our finding that ex vivo drug sensitivity provided prognostic information for PFS points to a possible impact of IPC on PFS when added to CRS. Still, it cannot be excluded that ex vivo drug sensitivity is only a prognostic marker reflecting tumor behavior unrelated to a therapeutic effect from IPC. The only way to differentiate between a purely prognostic vs predictive impact from ex vivo drug sensitivity assessment would be a clinical trial in which IPC is guided by ex vivo drug sensitivity data.

This would be a way to try to improve the effect of IPC by individualized selection of active drug(s) but also to decrease treatment related toxicity by avoiding IPC if no drugs seem active. The large interindividual sample differences in drug sensitivity that we observed clearly point to the potential for IPC individualization. Such trial would be technically feasible, because tumor tissue for ex vivo analysis could be obtained by laparoscopy before the CRS and ex vivo drug sensitivity data can be obtained within a few days. Such study is presently under discussion at our center.

Given that standard protocol IPC is currently part of the standard treatment for PMP, which conclusions can be drawn from the current study? The PMP samples in this study were essentially equally drug sensitive as peritoneal metastasis samples of colorectal cancer analyzed ex vivo with the same technique.^15^ Because several of the drugs analyzed are active in the treatment of colorectal cancer, similar drug activity also could be expected in PMP, provided similar drug exposure as in IPC.

The IC_50_ values for cisplatin and oxaliplatin were almost identical in the PMP subgroups. Given that a considerably higher dose of oxaliplatin compared with cisplatin can be given in IPC, the platinum of choice for IPC in PMP to achieve maximum effect is suggested to be oxaliplatin.

There were no major differences in drug sensitivity between the histopathological PMP subtypes, indicating that IPC protocols for PMP do not need to consider histopathological subtype. Furthermore, the frequently observed poor prognosis of PMCA compared with DPAM seems related to tumor biological factors other than tumor cell drug sensitivity.

The large interindividual differences in sensitivity to drugs used in IPC among the PMP samples were substantial. A reasonable interpretation is that IPC may be a more or less futile treatment step for patients with drug resistant tumor cells and that these patients would be better off with CRS alone.

Finally, tumor cells from patients previously exposed to cytotoxic drugs were generally more drug resistant than those previously unexposed. This is in line with the clinical observation that prior chemotherapy was associated with impaired prognosis in PMP.^5^ Because systemic chemotherapy seems not very active in PMP, it might be argued that PMP patients should go directly to CRS and HIPEC rather than be started on systemic chemotherapy with the risk for disease progression and development of drug resistance.^6^

## Conclusions

Ex vivo assessment of tumor cell sensitivity to cytotoxic drugs provides prognostic information in PMP and may be useful for sparing the most resistant patients from IPC expected to be futile. However, whether selection of drugs for IPC in PMP based on ex vivo assessment also is predictive for a treatment effect, and thus could be used for treatment individualization, needs to be investigated in a prospective, clinical trial.

## Electronic Supplementary Material

Supplementary material 1 (DOCX 28 kb)

Supplementary material 2 (DOCX 69 kb)
