# Supplementary material for: Drug Sensitivity Testing in Cytoreductive Surgery and Intraperitoneal Chemotherapy of Pseudomyxoma Peritonei
Source: Ann Surg Oncol. 2015 Jul 21;22:810–6. doi: 10.1245/s10434-015-4675-0 (PMC4686558; doi:10.1245/s10434-015-4675-0)
Supplement: Supplementary file 1 — Supplementary material 1 (DOCX 28 kb) [file 10434_2015_4675_MOESM1_ESM.docx]

**Supplementary Table 2. Univariate and multivariable Cox regression model for overall survival according to dichotomized IC_50_ drug sensitivity values (n = 92).**

|  |  | Univariate  HR (95% CI) | p | Mulivariate  HR (95% CI) | p |
| --- | --- | --- | --- | --- | --- |
| Oxaliplatin^a^ | Sensitive or not | 0.90 (0.42 - 1.93) | 0.8 |  |  |
| 5-FU^a^ | Sensitive or not | 1.20 (0.58 - 2.50) | 0.7 |  |  |
| Mitomycin C^a^ | Sensitive or not | 0.71 (0.33 - 1.56) | 0.4 |  |  |
| Doxorubicin^a^ | Sensitive or not | 0.69 (0.32 - 1.49) | 0.4 |  |  |
| Irinotecan^a^ | Sensitive or not | 0.84 (0.40 - 1.78) | 0.7 |  |  |
| Cisplatin^a^ | Sensitive or not | 0.64 (0.29 - 1.40) | 0.3 | 0.71 (0.32 – 1.58) | 0.5 |
| KPS index | < 90 vs. 90-100 | 7.93 (3.44 - 18.26) | 0.001 | 5.13 (2.05 – 12.86) | 0.001 |
| Histopathological subtype | DPAM | 1 |  | 1 |  |
|  | Hybrid | 1.17 (0.26 – 5.24) | 0.9 | 2.15 (0.45 – 10.35) | 0.4 |
|  | PMCA | 2.28 (1.06 – 4.88) | 0.05 | 1.24 (0.56 – 2.75) | 0.6 |
| PCI score | 21-39 vs. 1-20 | 4.33 (1.03 – 18.26) | 0.05 |  |  |
| Complete cytoreductive surgery^b^ | Yes vs. no | 11.73 (4.46 – 30.85) | 0.001 | 7.38 (2.62 – 20.76) | 0.001 |

^a^Below vs. above the median IC_50_ value. Low IC_50_ indicates better sensitivity. PCI score was not added to the final multivariable analysis because of colinearity with complete cytoreductive surgery. ^b^CC 0 – 1.
