# Supplementary material for: Drug Sensitivity Testing in Cytoreductive Surgery and Intraperitoneal Chemotherapy of Pseudomyxoma Peritonei
Source: Ann Surg Oncol. 2015 Jul 21;22:810–6. doi: 10.1245/s10434-015-4675-0 (PMC4686558; doi:10.1245/s10434-015-4675-0)
Supplement: Supplementary file 2 — Supplementary material 2 (DOCX 69 kb) [file 10434_2015_4675_MOESM2_ESM.docx]

Supplementary Table 1. IC_50_ values (μM, mean and standard deviation) for the indicated drugs in the pseudomyxoma peritonei samples investigated and divided into low and high grade according to Bradley et al. N indicates number of samples of each type. None of the differences observed were statistically significant.

| **Drug** | **Bradley** | **N** | **Mean**  **IC_50_** | **Std. deviation** |
| --- | --- | --- | --- | --- |
| Mitomycin | low grade | 59 | 21,5 | 58,1 |
|  | high grade | 26 | 18,8 | 20,2 |
| Doxorubicin | low grade | 58 | 2,46 | 4,79 |
|  | high grade | 24 | 1,46 | 2,62 |
| Cisplatin | low grade | 58 | 28,7 | 31,4 |
|  | high grade | 25 | 39,8 | 44,1 |
| Irinotecan | low grade | 59 | 339 | 616 |
|  | high grade | 26 | 230 | 282 |
| 5-FU | low grade | 60 | 590 | 431 |
|  | high grade | 26 | 658 | 382 |
| Oxaliplatin | low grade | 59 | 30,2 | 31,3 |
|  | high grade | 26 | 44,8 | 38,8 |
